# Human antibodies neutralizing the alpha-latrotoxin of the European black widow

**DOI:** 10.3389/fimmu.2024.1407398

**Published:** 2024-06-12

**Authors:** Maximilian Ruschig, Jana Nerlich, Marlies Becker, Doris Meier, Saskia Polten, Karla Cervantes-Luevano, Philipp Kuhn, Alexei Fedorovish Licea-Navarro, Stefan Hallermann, Stefan Dübel, Maren Schubert, Jeffrey Brown, Michael Hust

**Affiliations:** ^1^ Departments of Biotechnology and Medical Biotechnology, Institute for Biochemistry, Biotechnology and Bioinformatics, Technische Universität Braunschweig, Braunschweig, Germany; ^2^ Faculty of Medicine, Carl-Ludwig-Institute of Physiology, Leipzig University, Leipzig, Germany; ^3^ Departamento de Innovación Biomédica, Centro de Investigación Científica y de Educación Superior de Ensenada (CICESE), Ensenada, Mexico; ^4^ YUMAB GmbH, Braunschweig, Germany; ^5^ PETA Science Consortium International e.V., Stuttgart, Germany

**Keywords:** *Latrodectus tredecimguttatus*, *Latrodectus mactans*, toxin neutralization assay, phage display, recombinant antibodies, latrodectism, envenomation, IgG

## Abstract

Poisoning by widow-spider (genus *Latrodectus*) bites occurs worldwide. The illness, termed latrodectism, can cause severe and persistent pain and can lead to muscle rigidity, respiratory complications, and cardiac problems. It is a global health challenge especially in developing countries. Equine serum-derived polyclonal anti-sera are commercially available as a medication for patients with latrodectism, but the use of sera imposes potential inherent risks related to its animal origin. The treatment may cause allergic reactions in humans (serum sickness), including anaphylactic shock. Furthermore, equine-derived antivenom is observed to have batch-to-batch variability and poor specificity, as it is always an undefined mix of antibodies. Because latrodectism can be extremely painful but is rarely fatal, the use of antivenom is controversial and only a small fraction of patients is treated. In this work, recombinant human antibodies were selected against alpha-latrotoxin of the European black widow (*Latrodectus tredecimguttatus*) by phage display from a naïve antibody gene library. Alpha-Latrotoxin (α-LTX) binding scFv were recloned and produced as fully human IgG. A novel alamarBlue assay for venom neutralization was developed and used to select neutralizing IgGs. The human antibodies showed *in vitro* neutralization efficacy both as single antibodies and antibody combinations. This was also confirmed by electrophysiological measurements of neuronal activity in cell culture. The best neutralizing antibodies showed nanomolar affinities. Antibody MRU44–4-A1 showed outstanding neutralization efficacy and affinity to *L. tredecimguttatus* α-LTX. Interestingly, only two of the neutralizing antibodies showed cross-neutralization of the venom of the Southern black widow (*Latrodectus mactans*). This was unexpected, because in the current literature the alpha-latrotoxins are described as highly conserved. The here-engineered antibodies are candidates for future development as potential therapeutics and diagnostic tools, as they for the first time would provide unlimited supply of a chemically completely defined drug of constant quality and efficacy, which is also made without the use of animals.

## 1 Introduction

Most spiders express venomous proteins (1), but only a few genera are able to puncture the human skin and are thereby considered venomous for humans (2). One of the two clinically most relevant families are widow-spiders (genus *Latrodectus* spp.) (3), which consist of 32 species and are found in all inhabited areas of the world. Furthermore, widow-spiders invade new niches with climate change and globalization (4). For example, in the U.S., the American Association of Poison Control Centers reported 1134 bites in 2022 (5). The associated illness is called latrodectism and is recognized as a neglected tropical illness (6, 7), which can be classified into three severity grades (8). Grade 1 envenomation can be asymptomatic or characterized by local pain around the area of the bite. Grade 2 envenomations include pain, which can spread regionally to areas apart from the site of the bite, and also include local diaphoresis. Lastly, grade 3 envenomations or systemic envenomations are characterized by generalized pain, diaphoresis and alteration of vital signs, like hypertension, tachycardia, nausea, vomiting and headache (8). The most striking symptom is severe and long-lasting pain observed in 50% of latrodectism cases, with an average duration of symptoms of two days (2).

The venom of widow-spiders is a complex mixture of molecules, but mainly contains three large species-specific latrotoxins plus a fourth small protein-fraction, latrodectins or low molecular weight peptides (LMWP), which are non-toxic on their own but thought to enhance the toxicity of the large latrotoxins (9–11). The vertebrate-specific protein is called alpha-Latrotoxin (α-LTX), which is a potent neurotoxin that is highly conserved between *Latrodectus* (*L.*) species. Garb and Hayashi found that α-LTX shares ≥ 94% nucleotide identity between *Latrodectus species* by analyzing 618 base pair (bp) fragments of 16 *Latrodectus* species and two closely related *Steatoda* species (12). α-LTX is a 130 kilo dalton (kDa) sized protein, which consists of a distinct three domain structure (wing, body and head). It contains 11–22 ankyrin repeats in body and head and three conserved cysteines in the wing domain (13–16). Cryogenic electron microscopy (cryo-EM) showed that *L. tredecimguttatus* α-LTX is a stable, asymmetric homodimer, which forms a C4-symmetrical tetrameric complex upon the presence of divalent cations (17). Three distinct receptors for α-LTX in neuronal cells were identified, such as Latrophilin, which are involved in synapse formation and mechanosensation (18, 19) and mediate both Ca^2+^-dependent and -independent mechanisms of envenomation (20, 21). The toxic function is independent of the binding to these receptors and is mediated by the insertion of α-LTX into the presynaptic membrane creating Ca^2+^-permeable pores, which induce extensive neurotransmitter release, and ultimately can lead to swelling and bursting of the nerve terminals (22, 23).

Clinical treatment of latrodectism is mainly symptomatic by administration of parental opioids combined with benzodiazepines (8). Use of animal-derived antivenom is the most efficient available treatment option currently available and can significantly shorten the duration of illness, especially in higher grade envenomations (8). Nevertheless, administration of equine antivenom has been documented in two fatal cases that included induced hypersensitivity reactions and anaphylactic shock (8, 24). This is presumably related to the foreign nature of equine-derived proteins in combination with patients’ predisposition to developing an allergic reaction and raised concerns about the safety of antivenom administration (8, 24, 25). Despite efforts to improve safety concerns for the administration of antivenom (25–27) only a small fraction of patients is treated (28) and the manufacturing companies showed declining interest in providing antivenom (26).

The administration of foreign proteins naturally imposes an inherent risk of allergic reactions and serum sickness (26, 29–31). Risk can be reduced, e.g. by skin testing (29); unfortunately, skin testing was also described to be imprecise in nearly 50% of tested patients in the context of treating snake envenomation by equine-derived antivenom (24, 32). In addition, the production of therapeutic agents in horses is not regulated by international guidelines or rules to ensure animal-welfare, with a few exceptions only (33). Furthermore, the extraction of spider venom is cost- and time-intensive (34). Whenever applicable, animal-derived products or procedures should be replaced with non-animal derived alternatives (35). Recombinant human antibodies could improve patients’ safety and would avoid the use of animal-derived products. Additionally, recombinant antibodies could also be used for diagnostics, as to our knowledge there is still no specific option for diagnostics of latrotoxin envenomation and it is exclusively done by patients’ history (3). In addition, recombinant antibodies are sequence-defined and will be produced in mammalian cell culture and thereby production is assured for the future with constant quality.

Antibody phage display (36, 37) is a commonly used *in vitro* technique for the generation of recombinant human antibodies for therapeutic purposes. Currently, 14 therapeutic antibodies generated by phage display are approved by the European Medicines Agency/US Food and Drug Administration (38). Antibody fragments, mainly the single chain fragment variable (scFv) or fragment antigen binding (Fab) format can be selected *in vitro* against – in theory – any molecule from human antibody gene libraries, which can be naïve or immune (patient-derived) libraries without the need of animal immunization (39). These antibody fragments are linked to the phage protein III (pIII) and are displayed on the surface of filamentous M13 phage particles. The phagemid packaged in the phage carries the corresponding antibody gene fragment (40), which leads to a coupling of genotype and phenotype. The antibody selection is performed *in vitro* in a process called “panning”. Subsequently, the selected antibodies can be recloned and produced in any kind of antibody format, such as IgG or scFv-Fc. Overviews on antibody generation by phage display against toxins and for therapeutic purposes are given by Roth et al. and Frenzel et al., respectively (41, 42).

In this work, we report the generation of human recombinant IgG antibodies against α-LTX for the development of an alternative to equine-derived treatments. Moreover, they could be used to cover an unmet need of a specific diagnostic tool for latrodectism (6), as there is no specific agent to doubtlessly diagnose latrodectism and differential diagnosis is purely based on patients’ history and the patient being aware of the bite (43–45). These antibodies were characterized for their efficacy to neutralize the toxin in different *in vitro* assays.

## 2 Methods

### 2.1 Reagents

Purified Alpha-Latrotoxin from *L. tredecimguttatus* was obtained from Alomone Labs (Jerusalem, Israel, #LSP-130). Full venom from *L. mactans* was obtained from Spider Pharm (Yarnell, Arizona, USA, SKU 108V:1L0005) and from Octolab (Veracruz, Mexico). AlamarBlue HS was obtained from Invitrogen (Carlsbad, California, USA, #A50101). All other reagents and solutions were obtained from Roth (Karlsruhe, Germany) or Sigma-Aldrich (St. Louis, Missouri, USA).

### 2.2 Antibody selection via phage display

The antibody selection was performed as described previously with slight adjustments (46, 47).

For panning in MTP, 4 µg of α-LTX was diluted in phosphate-buffered saline (PBS) and immobilized on High Binding 96 well plates (Corning, Costar) overnight at 4°C. Next day the plates were blocked with 330 µL of 2% Panningblock (1% skim milk powder, 1% BSA in PBS with 0.05% Tween20) for 1 h at room temperature (RT) and then washed three times with ddH_2_0-T (ddH_2_0 _+_ 0.05% Tween20). Prior to incubating the library on coated antigen, the library (HAL9/10 separately with 1*10^11^ colony-forming units (cfu) each, or HAL9/10 mixed with 5*10^10^ cfu per library) was incubated on 2% panningblock for 1 h at RT, in order to deprive the library of unspecific/sticky phage (preclearance). Afterwards the library-mix was transferred to the coated antigen and incubated for 2 h at RT and subsequently washed 10 times. Bound phages were eluted using trypsin (10 µg/mL) at 37°C for 30 min and used for the next panning round. The phage solution was transferred to a 96 deep well plate (Greiner Bio-One), mixed with 150 µL *E. coli* TG1 (OD_600_ = 0.5) and incubated at 37°C for 30 min, followed by 30 min incubation with 650 rpm shaking, to facilitate the phage infection of *E. coli.* 1 mL of 2xYT-GA (1.6% (w/v) Tryptone; 1% (w/v) Yeast extract; 0.5% (w/v) NaCl (pH 7.0), 100 mM D-Glucose, 100 µg/mL ampicillin) was added and incubated for 1 h at 37°C, 650 rpm (OD_600_ = ~0.5). Afterwards, 1*10^10^ cfu M13K07 helper phage was added and again incubated 30 min at 37°C followed by 30 min at 37°C and 650 rpm shaking to facilitate phage infection of *E. coli*. The infected bacteria were pelleted at 3220xg for 10 min, supernatant was discarded and the pellet resuspended in 2xYT-AK (1.6% (w/v) Tryptone; 1% (w/v) Yeast extract; 0.5% (w/v) NaCl (pH 7.0), 100 µg/mL ampicillin, 50 µg/mL kanamycin). Phage amplification was done overnight at 30°C, 650 rpm shaking, and the amplified phage were used for the next panning round. In total, five rounds of panning were performed, while increasing washing stringency (10x round 1, 20x round 2, 30x round 3, 4 and 5) and reducing antigen availability (4 µg round 1, 2 µg round 2, 1 µg round 3, 4 and 5). In the fourth panning round, washing and amount of antigen was kept constant and the fourth and fifth rounds were conducted mainly to increase phage amplification, without increasing the selection pressure. After the fifth panning round, single clones from panning rounds three, four and five were analyzed for production of anti-α-LTX specific scFv by screening ELISA.

For panning in solution and Strep-captured panning, α-LTX was biotinylated using EZ-Link Sulfo-NHS-LC-Biotin kit (Thermo Fisher Scientific) according to manufacturer’s instructions. α-LTX was dialyzed against PBS. Similarly to panning in MTP, in a first step, libraries (1*10^11^ cfu of HAL9/HAL10 separately) were precleared of unspecific phage by preincubating library on 2% BSA in one well of a MTP for 45 min at RT. Afterwards, a second preincubation step on magnetic Streptavidin beads (Dynabeads M-280 Streptavidin, Invitrogen) was performed in solution for 45 min at RT rotating. The supernatant containing the precleared library was separated using a magnetic stand and 100 ng biotinylated α-LTX added and rotated for 2 h at RT. Bound phage were extracted by adding Streptavidin beads and incubation was done for 30 min at RT under rotation. Unbound phages were washed 10x times with PBS supplemented with 0.05% Tween20 (PBST) by separating beads on a magnetic stand and discarding the supernatant. Afterwards bound phages were eluted and panning continued as described for panning in MTP. In total, three rounds of panning were performed.

For Strep-captured panning, two wells of an MTP were coated with 2 µg/mL Streptavidin and wells blocked with 330 µL 2% Panningblock for 1 h at RT. HAL9/10 were mixed (5*10^10^ cfu per library) and preincubated on Streptavidin, while α-LTX was captured on a second Streptavidin well for 1 h at RT. Precleared library mix was transferred to antigen and panning done for 2 h at RT. To reduce selection of Streptavidin-specific phage further, 5 µg Streptavidin was added to the panning well as soluble competition. After washing, panning was continued as described above. In total, five rounds of panning were performed.

### 2.3 Production of soluble, monoclonal scFv in MTP and screening ELISA

Monoclonal, soluble antibody fragments (scFv) were produced in 96 well polypropylene MTPs (U96PP, Greiner Bio-One) as described before (46, 48). In brief, 180 µL of 2xYT-GA were inoculated with single colonies bearing the scFv-expressing phagemids and incubated overnight at 37°C, 800 rpm in a MTP shaker (VorTemp 56™ Shaking Incubator, LabNet, Edison, NJ, USA). Next day, 170 µL 2xYT-GA were inoculated with 10 µL of the overnight culture and grown at 37°C, 800 rpm for 2 h (approx. OD_600nm_ 0.5). Bacteria were pelleted at 3220xg for 10 min and supernatant was discarded. To induce expression of antibody genes, pellets were resuspended in 180 µL 2xYT-A supplemented with 50 µM of isopropyl-beta-D-1-thiogalactopyranoside (IPTG) and incubated overnight at 30°C, 800 rpm. Next day, bacteria were pelleted at 3220xg, 4°C for 20 min and supernatant was used in screening ELISA.

For the ELISA, 100 ng/well of antigen were coated in PBS overnight at 4°C in High Binding 96 well plates (Corning, Costar). The next day, plates were blocked using 2% milk powder diluted in PBST (MPBST) for 1 h at RT and plates were washed three times using ddH_2_O with 0.05% Tween20. 40 µL supernatant, containing the secreted scFv, was mixed with 60 µL 2% MPBST and incubated on the antigen for 2 h at RT. Subsequently, the plates were washed again three times using ddH_2_O with 0.05% Tween20. Bound scFv were detected using murine mAb 9E10 (diluted 1:50 in 2% MPBST), which recognizes the C-terminal myc-tag and a secondary goat-anti-mouse serum conjugated with horseradish peroxidase (HRP) (A0168, Sigma-Aldrich, 1:42,000 dilution in 2% MPBST), each incubated for 1 h at RT, followed by three washing steps using ddH_2_O with 0.05% Tween20. Bound antibodies were visualized with tetramethylbenzidine (TMB) substrate [20 parts TMB solution A (30 mM Potassium citrate; 1% (w/v) Citric acid (pH 4.1)] and 1 part TMB solution B [10 mM TMB; 10% (v/v) Acetone; 90% (v/v) Ethanol; 80 mMH_2_O_2_ (30%)] were mixed). After stopping the reaction by addition of 1 N H_2_SO_4_, absorbance at 450 nm with a 620 nm reference was measured in an ELISA plate reader (Epoch, BioTek). Monoclonal binders were sequenced and analyzed using VBASE2 (www.vbase2.org) (49) and possible glycosylation positions in the complementarity-determining regions (CDRs) were analyzed (50).

### 2.4 Subcloning of scFv into expression vectors for Expi293F suspension cell production

Selected scFv fragments were produced in mammalian cell culture in Expi293F cells. For this purpose, scFv gene fragments were subcloned into feasible expression vectors. In a first step, scFv fragments were subcloned into pCSE2.7-hFc-IgG (48) using NcoI/NotI (NEB). Additionally, expression vectors for production of hIgG1 and hFab antibodies were constructed. Here, VH and VL were cloned separately into pCSEHh1c-XP (heavy chain) and pCSL3hλ-XP/pCSL3hκ (light chain lambda/kappa) for IgG (51) and into pCSE2.5-Hc-hFab.2-XP (heavy chain) and pCSLCl-hFab.2-XP/pCSLCk-hFab.2-XP (light chain lambda/kappa) for production of hFab respectively via Golden Gate Assembly using Esp3I restriction enzyme (New England Biolabs, Frankfurt, Germany). Expi293F cells were cultured in GIBCO FreeStyle F17 expression media (Thermo Fisher Scientific) supplemented with 0.1% Pluronic F68 and 8 mM L-Glutamine (PAN Biotech) at 37°C, 110 rpm and 5% CO_2_. Transfection was carried out at cell densities between 1.5 – 2*10^6^ cells/mL and over 90% viability. DNA: PEI complexes were formed with 1 µg DNA/mL transfection volume (1:1 ratio of vector for IgG or Fab production) and 5 µg/mL transfection volume 40 kDa polyethylenimine (PEI) (Polysciences). Here, DNA and PEI were first diluted separately in supplemented F17 media in 5% transfection volume, then mixed thoroughly and incubated for 25 min at RT before adding into the cells. 48 h post transfection cells were fed by adding the same volume of HyClone SFM4Transfx-293 media (GE Healthcare) supplemented with 8 mM L-Glutamine. Additionally, cells were supplemented with HyClone Boost 6 (GE Healthcare) adding 10% of culture volume. One week post transfection, culture supernatant was harvested by centrifugation at 1500xg for 15 min and purified using Protein-A purification.

### 2.5 Protein-A purification

Purification was done as described previously (52). Small-scale production was purified with 24 well filter plates with 0.5 mL resin. Larger scale productions (above 15 mL) were purified with 1 mL Mab-Select SuRe or HiTrap Fibro PrismA columns (Cytiva) on Äkta Go or Äkta Pure (Cytiva) or using the Profinia System (BIO-RAD). His-tagged proteins were purified using HisTrap FF Crude columns (Cytiva). All purifications were done according to manufacturer’s instructions.

### 2.6 SDS-PAGE and immunoblot analysis

Samples were diluted in PBS supplemented with only Laemmli-buffer (non-reducing) or containing beta-mercaptoethanol (reducing) and boiled for 10 min at 56°C (non-reducing) or 95°C (reducing). Samples were cooled down and 1–2 µg of protein was separated with 12% SDS-PAGE at 180 V for 45–75 mins. Electrophoresis of venom samples was done by using two different separation gels, as black widow spider venom contains very small as well as larger proteins. Small proteins were separated in the lower 12% gel, while the large latrotoxins were separated in the upper 8% gel. SDS-PAGE was stained with Coomassie Brilliant Blue and destained with 10% acetic acid.

For Immunoblot analysis, separated proteins were transferred onto a polyvinylidene fluoride (PVDF) membrane using the TransBlot Turbo transfer device (BIO-RAD). Afterwards membranes were blocked with 2% MPBST for 1 h at RT. Primary antibodies were diluted to 10 µg/mL in MPBST and incubated for 1 h at RT. Binding of antibodies to venom proteins was detected using goat-anti-human IgG (Fc-specific, A0170 Sigma-Aldrich) conjugated to horse radish peroxidase (HRP) in a final dilution of 1:70,000. As positive control, polyclonal anti- α-Latrotoxin produced in rabbit (L1913, Sigma-Aldrich) diluted to 1:50 was used and binding detected using donkey anti-rabbit IgG (711–035-152, Jackson ImmunoResearch) conjugated to HRP in a final dilution of 1:20,000.

### 2.7 Titration ELISA

For titration ELISA, *L. tredecimguttatus* α-LTX or *L. mactans* venom was immobilized with 1 µg/mL or 2 µg/mL diluted in PBS, respectively in High binding 96 well MTP (Corning) at 4°C overnight. Next day, plates were blocked using 2% MPBST for 1 h at RT and plates washed subsequently using ddH_2_0-Tween. Antibodies were titrated from 100 nM to 0.001 nM for α-LTX titration ELISA and from 310 nM to 0.0031 nM for venom titration ELISA and incubated for 1 h at RT and plates washed again. Detection of binding was done using HRP conjugated goat-anti-human IgG (Fc-specific, A0170, Sigma-Aldrich, final dilution 1:70,000) and plates were washed subsequently. TMB was used as substrate and ELISA developed for 15 min before reaction was stopped using 1 N H_2_SO_4_. Plates were measured using at 450 nm with 620 nm as reference wavelength.

For epitope binning, MRU44–4-A1 was used as a capture antibody in Fab format and High binding 96 well MTP (Corning) coated with 2 µg/mL diluted in PBS overnight at 4°C. Next day, plates were blocked using 330 µL 2% MPBST and incubated 1 h at RT and afterwards plates washed using ddH_2_0-Tween. *L. tredecimguttatus* α-LTX was diluted to 1 µg/mL in 2% MPBST and incubated on capture antibody for 1 h at RT and plates subsequently washed again. Antibodies were titrated from 10 µg/mL to 0.1 µg/mL in 2% MPBST and incubated on α-LTX for 1 h at RT. After plates were washed, detection of bound IgG was done using HRP conjugated goat-anti-human IgG (Fc-specific, A0170, Sigma-Aldrich, final dilution 1:70,000) and plates washed a final time. TMB was used as a substrate and ELISA was developed for 15 min before reaction was stopped using 1 N H_2_SO_4_. Plates were measured using an ELISA reader (Epoch, BioTek) at 450 nm with 620 nm as reference wavelength.

### 2.8 Establishment of novel *in vitro* α-LTX neutralization assay

For screening of α-LTX neutralization and selection of lead antibodies, a functional assay was established using the neurosecretory cell line PC-12 and alamarBlue cell viability reagent. PC-12 cells were grown in RPMI1640 medium (Capricorn Scientific) supplemented with 5% fetal bovine serum (Merck) and 10% donor horse serum (Biochrom) at 37°C and 5% CO_2_. In a first step, α-LTX dependent activity on PC-12 cells inducing pathogenic effects was validated. Different assay parameters were tested (number of cells per well, time of intoxication, time of alamarBlue development, α-LTX concentration) (data not shown). Finally, a protocol was established intoxicating 20,000 cells/well with α-LTX starting at a concentration of 50 nM titrated down to 0.05 nM. Intoxication was done for 15 h at 37°C and 5% CO_2_. All media were spiked with varying CaCl_2_ concentrations (0, 1, 5 and 10 mM) and after intoxication, 10% (v/v) of alamarBlue was added to the culture volume. Assay was developed for 6–8 h at 37°C and 5% CO_2_. α-LTX-induced pathogenic effects were determined by measuring relative fluorescent units (RFU) at 595 nm with 555 nm excitation wavelength using a Tecan Spark multimode plate reader. Mock intoxication controls, treating the cells with medium without containing any α-LTX and α-LTX controls without adding any antibody were included.

### 2.9 High-throughput screening of IgG for α-LTX neutralization

For screening of α-LTX neutralization, a mean α-LTX concentration of 4.2 nM was chosen. All media were spiked with 10 mM CaCl_2_. Antibodies were prepared in 50-fold molar excess to α-LTX with 210 nM per antibody and titrated to 0.067 nM and preincubated with toxin for 1 h at RT. Afterwards 20,000 PC-12 cells/well were intoxicated with preincubation mix for 15 h at 37°C, 5% CO_2_. Assay was developed by adding 10% (v/v) alamarBlue to the cells and incubating for 6–8 h at 37°C, 5% CO_2_. Cell viability was determined by measuring RFU at 595 nm with 555 nm excitation using a multimode plate reader (Spark, Tecan). All antibodies were tested in duplicates. Due to the multitude of antibodies, screening was done in three individual experiments. STE90-C11 was included as unrelated isotype control. MRU44–4-A1 was included in each experiment as internal positive control. Furthermore, controls intoxicating the cells without any antibody and mock intoxicating the cells with media not containing any α-LTX were included.

### 2.10 Biolayer interferometry

Antibody affinity of MRU44–4-A1 to α-LTX was measured in different antibody formats using Bio-layer interferometry (BLI) with the Octet qKe (Fortebio/Sartorius GmbH, Göttingen, Germany). IgG and scFv-Fc were measured using ProA biosensors, while Fab format was measured using FAB2G sensor. Baseline measurement was performed in assay buffer (TBS supplemented with 1% BSA and 0.05% Tween20) for 60 s. Subsequently, antibodies were loaded onto the sensors for 180 s in assay buffer with 10 µg/mL of antibody. Measurements were done in presence or absence of 10 mM CaCl_2_ to induce oligomerization of α-LTX. After establishing a stable baseline of loaded antibodies, association of α-LTX was measured for 300 s in dilution series from 158 nM to 0.5 nM. A control without α-LTX and a control with unloaded sensor was included. Following association, dissociation of α-LTX was measured for 600 s by transferring the sensors to respective assay buffer (in presence or absence of 10 mM CaCl_2_). Analysis was done by subtracting recorded baseline measurements (0 nM α-LTX) and modeling of binding kinetics was done using a global 1:1 binding model.

### 2.11 Lyophilization and QC of lyophilized anti-α-LTX lead candidates

Lyophilization was done as described in (53). For buffer formulation, 10 mM sodium phosphate buffer (pH 7.4) supplemented with 2% Trehalose was used and antibodies concentration set to >1 mg/mL. IgG were lyophilized as 250, 500 or 1000 µL vials in 11 mm crimp-glass vials. Vials were loosely closed with lyophilization caps to ensure water evaporation and prefrozen at -80°C. Lyophilization was performed using Alpha2–4 LSCplus (Martin Christ) and shelf precooled to -80°C. Primary drying was done at -50°C and vacuum of 0.1 mbar for 40 min before temperature was raised to -35°C, while vacuum was kept constant for in total 37 h. For final drying of samples, temperature was increased to 20°C and vacuum lowered to 0.08 mbar for in total 36 h. Subsequently chamber was flooded with nitrogen and lyophilization caps closed tightly with height-adjustable table and vials finally closed with metal caps. Reconstitution was done using ddH_2_O using lyophilized volume and antibodies quality controlled in size exclusion chromatography and *in vitro* neutralization assay.

### 2.12 Mouse cortical cultures

Neocortical neuronal cultures from P0 to P1 mice were prepared similarly as previously described (54). Briefly, mice were decapitated and cerebral cortices were removed, dissected and enzymatically digested with Papain (Sigma) in the presence of DNAse (Sigma), followed by mechanical dissociation and centrifugation through a cushion of 4% bovine serum albumin (Sigma). These steps were completed using Hibernate medium (ThermoFisher). Cells were then plated onto Poly-L-Lysine (Sigma) coated coverslips in 24 well plates. For each coverslip, 35,000 cells were allowed to settle in a 40 μL drop for about 30 min and then each well was filled with 500 μL growth medium: NeurobasalA/B27 (Invitrogen) supplemented with GlutaMax (0.25%, Invitrogen), Glutamine (0.25–0.5 mM, Sigma), Penicillin/Streptomycin (1:100, ThermoFisher) and heat-inactivated fetal calf serum (10%, Sigma). Medium was partially exchanged on day 3 (800 μL) and day 14 (500 μL) with fresh maintenance medium: BrainPhys (StemCell), B27 (2%, Invitrogen), GlutaMax (0.25%, Invitrogen), Penicillin/Streptomycin (1%, ThermoFisher). Cultures were maintained for up to 3 weeks at 37°C and 5% CO_2_.

### 2.13 Electrophysiology

Postsynaptic voltage-clamp recordings were performed in mouse cortical neurons [days *in vitro* (DIV) 14–17] using a HEKA EPC10 amplifier (HEKA Elektronik, Lambrecht/Pfalz, Germany). Pipette solution for voltage clamp recordings contained (in mM): 130 CsMeSO_3_, 10 TEA-Cl, 10 HEPES, 5 EGTA, 3 Mg-ATP, 0.3 Na-GTP, 5 Na-Phosphocreatin, 3 QX314-Cl, pH adjusted with CsOH to 7.32 and osmolarity adjusted by sucrose to 300 mOsm. Series resistance (Rs) was on average 9.4 ± 0.3 MΩ (n=32, mean ± SEM) and was compensated to a remaining Rs of 4.7 ± 0.2 MΩ. Pipettes were pulled from borosilicate glass (Science Products, Hofheim, Germany) with a DMZ Universal Electrode (Zeitz Instruments, Martinsried, Germany) with resistance of 3 - 4 MΩ.

Recordings of the holding current (I_hold_) and spontaneous miniature excitatory postsynaptic currents (mEPSCs) were performed at a holding potential of -70 mV in an extracellular recording solution containing (in mM): 145 NaCl, 2.5 KCl, 1.2 MgCl_2_, 2 CaCl_2_, 10 HEPES, 10 glucose, pH adjusted by NaOH to 7.4. To isolate mEPSCs the extracellular solution was supplemented by 1 µM tetrodotoxin (TTX) to block action potential induced synaptic transmission and 10 µM SR95531 to block GABAA receptors. The voltage was not corrected for a calculated junction potential of 9.6 mV. Recordings were performed at RT.

After establishing a stable baseline recording period (4 minutes), preincubated extracellular solution supplemented by either 1 nM α-LTX alone or 1 nM α-LTX and 10 nM of the respective antibody was washed-in through the perfusion system and I_hold_ and mEPSCs were monitored up to 20 minutes after wash-in. Spontaneous mEPSC were detected with the waveform template matching algorithm of the NeuroMatic plug-in (Version 3) for Igor Pro (WaveMetrics, Lake Oswego, OR, USA; Version 9). For each cell, the mEPSC frequency was calculated for every minute and normalized to the mean mEPSCs frequency before wash in of α-LTX in the presence of the respective antibody. The onset time of the toxic effect was set as the time when the mEPSC frequency exceeded 4xSD of the mean mEPSC frequency before wash-in.

Experimenter was blind to treatment and blinding was performed by an independent investigator. Unblinding of the experimental groups was performed after analysis and disclosure of the data by the experimenter.

The differences between the effect of α-LTX in the presence of the antibodies were tested by one-way ANOVA followed by Bonferroni’s *post-hoc* comparisons tests. Calculations of statistical tests were performed with jamovi (https://www.jamovi.org).

## 3 Results

### 3.1 Antibodies against α-LTX were selected by phage display

In order to develop therapeutic antibodies against α-LTX, phage display panning of naïve libraries HAL9/10 was performed on *L. tredecimguttatus* α-LTX. Various panning approaches were employed in order to find the most efficient strategy (**Table 1**).

**Table 1:** Unique scFv (by sequence) that bound α-LTX per number of positive hits per number of clones analyzed in α-LTX ELISA.

Library	α-LTX coated MTP	Captured α-LTX	α-LTX in Solution
**HAL9 (kappa)**	5/23/184	–	0/0/92
**HAL10 (lambda)**	0/0/184	–	0/0/92
**Library Mix (HAL9+HAL10)**	69/213/1336	1/3/276	–

MTP, multitier plates, Captured: biotinylated α-LTX was immobilized on MTP coated with Streptavidin, Panning in solution was done by incubating the libraries with biotinylated α-LTX in solution, to be captured after 60 minutes on MTP coated with Streptavidin.

In total, 75 unique antibodies binding *L. tredecimguttatus* α-LTX were selected after up to five panning rounds with an overall hit ratio of 15.3% and a ratio of 4.8% individual antibodies. Subsequently, 53 antibodies were subcloned and successfully produced as human IgG. The remaining 22 antibodies failed to be recloned or produced as IgG. "-" = not performed.

### 3.2 Quantification of latrotoxin activity on PC-12 cells by alamarBlue cell viability assay

**Figure 1:**
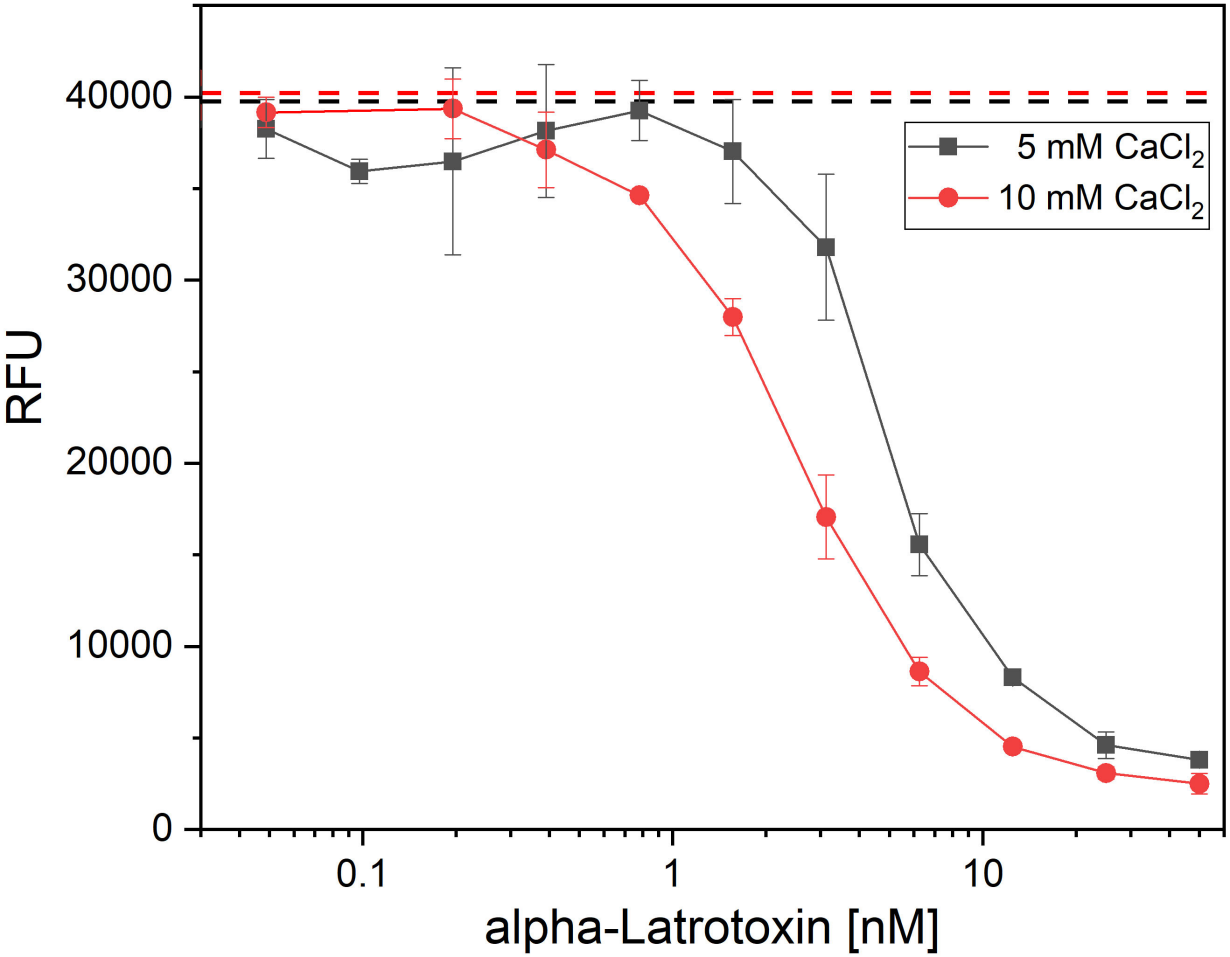
Latrotoxin activity assay in PC-12 cells using alamarBlue. Cytotoxic effect of α-LTX on PC-12 cells was validated by intoxicating 20,000 cells/well for 15 h with different α-LTX concentrations starting at 50 nM diluted to 0.05 nM. All media were spiked with either 5 mM (black line) or 10 mM (red line) CaCl_2_. Mean value of control, treating cells with medium and buffer not containing α-LTX is shown in black and red dashed lines. After intoxication,10% (v/v) alamarBlue was added to the cells and after 4–15 h development emission was measured at 595 nm using Tecan Spark with 555 nm excitation. Error bars represent the standard deviation of two measurements.

As the available assays which so far used in α-LTX research were not providing the necessary combination of high-throughput compatibility, safety and sensitivity, a new assay was developed to screen the antibodies for α-LTX neutralization, α-LTX induces Ca^2+^-dependent, cytotoxic effects on the neurosecretory PC-12 cell line (55). AlamarBlue is a non-toxic, cell viability reagent, which can be used to monitor oxidation in metabolically active cells, and thereby can be used quantitatively for cell viability assays. In a first step to establish a novel α-LTX neutralization assay, the concentration-dependent activity of α-LTX on PC-12 cells using alamarBlue was verified. In preliminary experiments, the number of cells per well, Ca^2+^ concentration, time of intoxication and time of assay development were optimized (data not shown) to measure cytotoxic effects of α-LTX on PC-12 cells in a toxin- and calcium concentration-dependent manner (**Figure 1**). A mean α-LTX concentration of 4.2 nM was chosen to ensure a 50-fold molar excess of antibody to toxin in the highest antibody concentration and to work in the lower end of the linear range of α-LTX activity curve. Always 20,000 cells per well were intoxicated for 15 h at 37°C and 5% CO2. All media were spiked with 10 mM CaCl_2_ and alamarBlue developed for 6–8 h at 37°C and 5% CO_2_ before RFU were determined in the Tecan Spark multimode plate reader at 555 nm excitation and 595 nm emission.

### 3.3 α-LTX neutralizing IgG

**Figure 2:**
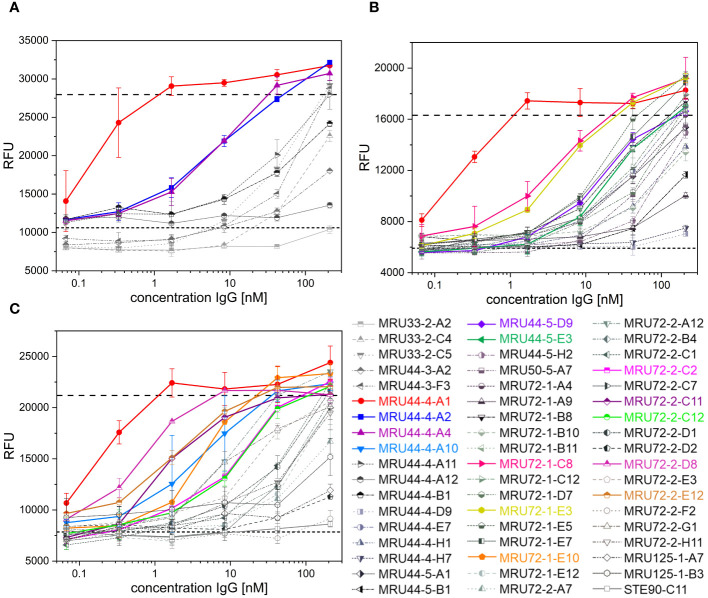
Latrotoxin neutralization and titration ELISA of lead IgGs. **(A-C)** Screening of α-LTX neutralization with 53 IgG against α-LTX on PC-12 cells. Antibodies were titrated in 210 nM – 0.07 nM and incubated with 4.2 nM α-LTX. Mean values of mock intoxication treating the cells without toxin is marked in long dashed lines, mean value of treating cells with α-LTX without adding antibodies is marked in short dashed lines. Lead IgG selected for further development are marked in colors. Measurements were prepared in duplicates and IC_50_ values were determined using OriginPro Hill1 non-linear fit. Screening was done in individual experiments and multiple plates per experiment are summarized in each graph. Error bars represent the standard deviation of two measurements.

**Figure 3:**
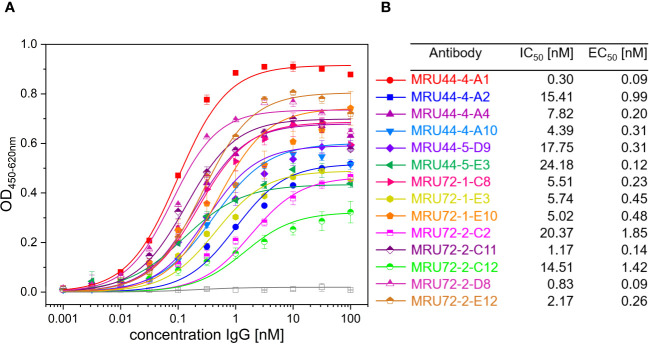
Titration ELISA of lead IgGs and IC_50_ and EC_50_ determined in screening of IgG and titration ELISA of selected lead IgGs. **(A)** Titration ELISA of 14 lead IgG on 1 µg/mL immobilized α-LTX. Antibodies were titrated using 100 nM – 0.001 nM antibody. STE90-C11 was included as unrelated isotype control. Measurements were prepared in duplicates and EC_50_ values determined using OriginPro Hill1 non-linear fit. **(B)** Summary of IC_50_ and EC_50_ values determined in **Figures 2**, **3** for selected lead IgG antibodies. Error bars represent the standard deviation of two measurements.

The alamarBlue/PC12 cell-based assay allowed to detect α-LTX neutralization by an antibody when it provided cell viability comparable to non-intoxicated control. An unrelated anti-SARS-CoV-2 IgG (STE90-C11) (52) was used as negative control. 45 out of 53 tested antibodies showed neutralization of α-LTX with varying efficacies, while 35 reached or exceeded viability levels of mock intoxication control (**Figure 2**). MRU44–4-A1 showed substantially better neutralization potency than all other tested antibodies and was in the following included in each experiment as an internal positive-control. The half maximal inhibitory concentrations (IC_50_) were determined to rank antibodies for their neutralization efficacy (**Figure 3B**). The 14 antibodies exhibiting the lowest IC_50_ (values ranging from 0.3 to 24 nM respectively) were selected for further development (highlighted in color in **Figure 2**).

The selected lead candidates were also tested in ELISA to determine the half maximal effective concentration (EC_50_) of antigen binding (**Figures 3A, B**). The strong neutralization efficacy of MRU44–4-A1 to α-LTX was in accordance with the sub-nanomolar EC_50_ of 0.09 nM measured by ELISA. In general, all lead candidates showed strong binding of α-LTX with EC_50_ ranging from 0.09 nM to 1.85 nM with a mean EC_50_ of 0.5 nM.

### 3.4 α-LTX neutralization on mouse cortical neurons

**Figure 4:**
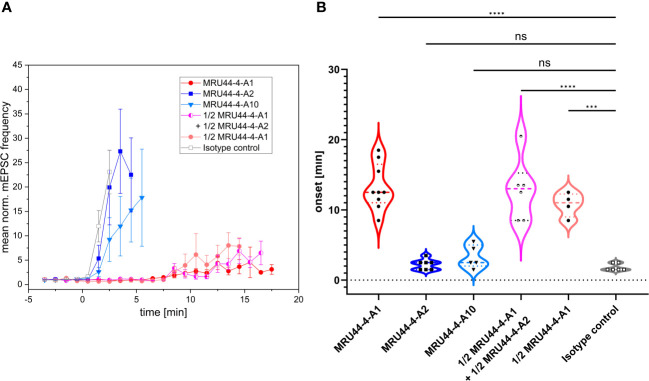
Effect of selected antibodies onto α-LTX-induced increase of vesicular release. **(A)** Mean normalized mEPSC frequencies in cultured cortical neurons before and in the presence of a mixture of α-LTX and different antibodies or antibody combination. Error bars represent the standard deviation of multiple measurements (MRU44–4-A1: n = 9; MRU44–4-A2: n = 7; MRU44–4-A10: n = 5; MRU44–4-A1 + MRU44–4-A2: n = 6; 1/2 MRU44–4-A1: n = 4; STE90-C11: n = 6). After establishing a stable baseline recording period (4 minutes) preincubated extracellular solution supplemented with 1 nM α-LTX and 10 nM of respective antibody was washed in through the perfusion system (start of wash-in at time point 0). For antibody combination 5 nM per antibody and for 1/2 MRU44–4-A1 only 5 nM antibody was used. **(B)** Mean and individual onset times of α-LTX-induced pathogenic effect. For each cell the onset time was set as time point when the mEPSC frequency exceeded 4xSD of the mean mEPSC frequency before wash-in. For MRU44–4-A1 and combination of MRU44–4-A1 + MRU44–4-A2 in three and one single cell measurement, respectively, mEPSC frequency did not reach 4xSD during recording time. In these cases, onset time was set to the time the recording ended. P values were calculated by comparison of respective onset times by one-way ANOVA followed by Bonferroni’s *post-hoc* comparisons tests. *** p=0.0001, **** p<0.0001, ns=not significant.

To test the neutralization efficacy of the antibodies onto an α-LTX-induced increase in neurotransmitter release (56), spontaneous miniature excitatory postsynaptic currents (mEPSCs) were measured in cultured murine cortical neurons by means of whole-cell patch clamp recordings. The mEPSC frequency was calculated every minute before and during bath application of a toxin-antibody mixture consisting of α-LTX and MRU44–4-A1, MRU44–4-A2, MRU44–4-A10, the isotype control STE90-C11 and a combination of MRU44–4-A1 + MRU44–4-A2, respectively (**Figure 4A**). The mEPSC frequency was normalized to the mean mEPSC frequency before wash-in (addition of toxin:antibody mixture) and averaged across all cells for every toxin-antibody mixture, respectively. The onset time of the α-LTX-induced pathogenic effect was defined as the time point in which the measured mEPSC frequency exceeds 4xSD of the mean mEPSC frequency before wash-in of the toxin-antibody mixture (**Figure 4B**). The onset thus reflects the delay until α-LTX-induced neurotransmitter release.

The isotype control STE90-C11 showed an α-LTX-induced increase in neurotransmitter release immediately after wash-in of the toxin-control antibody mixture, confirming the fast pathogenic effect of α-LTX. In comparison, MRU44–4-A1 significantly delayed the onset of α-LTX effect up to 18 min after wash in. In three out of 9 single cell measurements, 4xSD was not reached during recording time and thereby no onset of the α-LTX effect could be determined. In these experiments, the onset time was set to maximum measurement time. MRU44–4-A2 and MRU44–4-A10 slightly delayed the onset of pathogenic α-LTX effects. The strong neutralization efficacy of MRU44–4-A1 shown in the alamarBlue based neutralization assay could be verified in cultured neurons showing a clear deceleration of the α-LTX-induced increase in neurotransmitter release. The combination of MRU44–4-A1 and MRU44–4-A2 did not improve neutralization over levels of MRU44–4-A1 (10 nM) used alone.

In general, MRU44–4-A2 and MRU44–4-A10 did not show a strong neutralization efficacy in electrophysiological recordings. To be noted, only a 10-fold molar excess of antibody to toxin was used in comparison to a 50-fold molar excess in the alamarBlue neutralization assay.

### 3.5 Epitope binning

**Figure 5:**
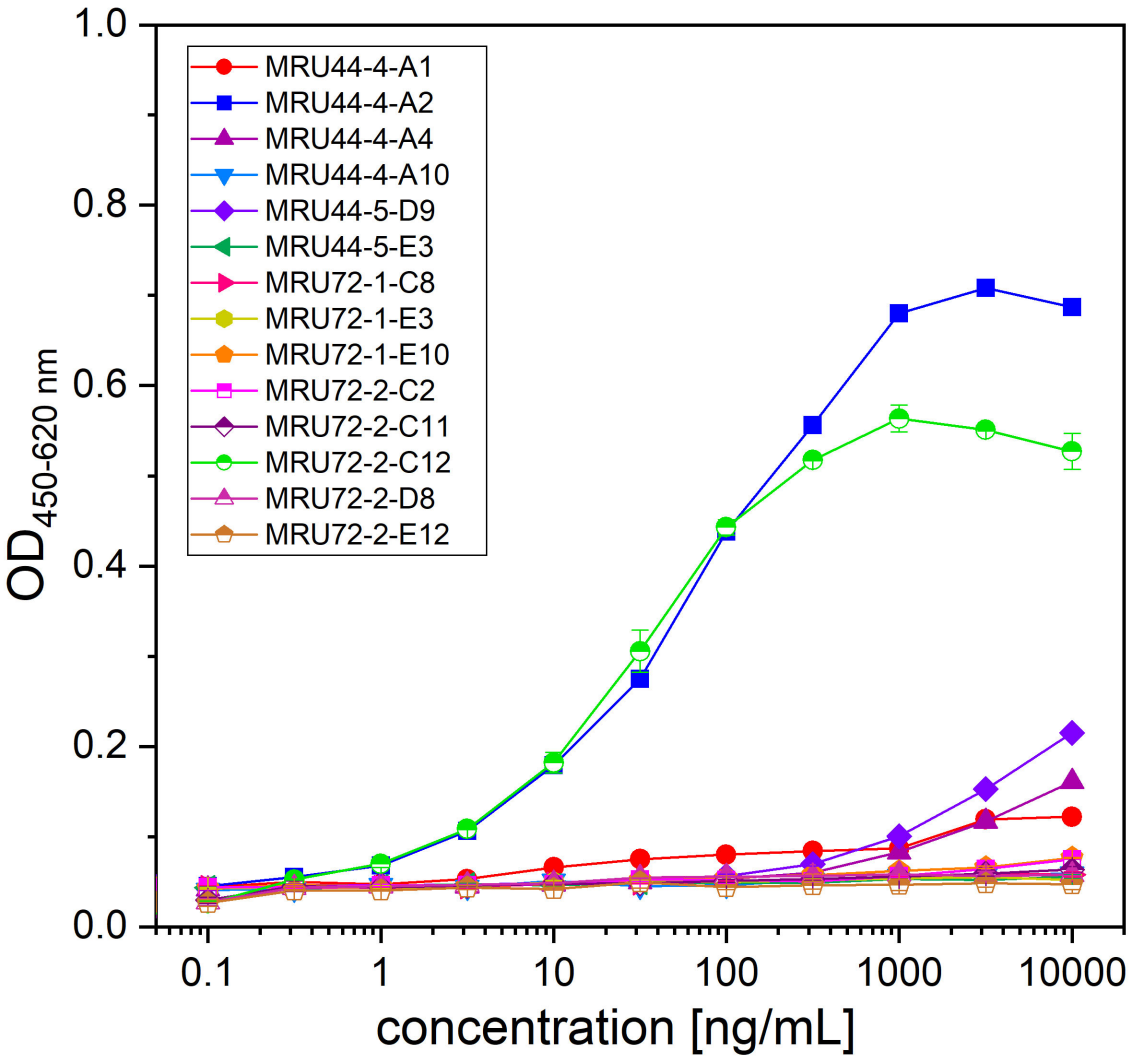
Epitope compatibility assessed by capture ELISA. MRU44–4-A1 in Fab format was immobilized in 96 well MTP with 2 µg/mL and plates blocked with 330 µL 2% MPBST. Lead IgG were titrated starting with 10 µg/mL diluted to 0.0001 µg/mL. Error bars represent the standard deviation of two measurements.

Using oligoclonal mixtures of antibodies can enhance neutralization efficacy of the individual antibodies by additive or synergistic effects and mimic the advantages of a polyclonal antibody serum, while still ensuring a defined product (48). To find antibodies which are binding to different epitopes and can be used in combination with MRU44–4-A1 in an oligoclonal antibody cocktail, this antibody was produced as human Fab. MRU44–4-A1 was chosen as lead candidate, as this antibody showed the highest affinity and neutralization efficacy (see **Figures 2**, **3**). Consequently, MRU44–4-A1 was used as capture antibody in the Fab format and 14 IgGs were titrated over captured α-LTX to analyze epitope compatibility (**Figure 5**). MRU44–4-A2 and MRU72–2-C12 were the only antibodies binding to α-LTX in the presence of MRU44–4-A1.

### 3.6 Affinity of different antibody formats of MRU44–4-A1

Binding affinity in scFv-Fc, IgG and Fab format of MRU44–4-A1 to α-LTX was measured using biolayer-interferometry (BLI) on monomeric/dimeric toxin, as well as tetrameric toxin, by using 2 mM ethylenediaminetetraacetic acid (EDTA) or 10 mM CaCl_2,_ respectively. This was done to elucidate if antibodies have a preference in binding the inactive (dimeric) or active form (tetrameric) oligomeric state of the toxin. Here, the dissociation constant (K_D_) was measured between 1.8 nM to 12.2 nM (**Table 2**).

**Table 2:** Biolayer-interferometry of MRU44–4-A1 in different antibody formats and IC_50_ determined in alamarBlue neutralization with different antibody formats.

Setup	Antibody format	K_D_ [nM]	IC_50_ [nM]
**α-LTX + 2 mM EDTA**	MRU44–4-A1 scFv-Fc	8.4	n.a.
	MRU44–4-A1 IgG	6.3	n.a.
	MRU44–4-A1 Fab	1.8	n.a.
**α-LTX + 10 mM CaCl_2_**	MRU44–4-A1 scFv-Fc	12.2	0.56
	MRU44–4-A1 IgG	4.2	0.35
	MRU44–4-A1 Fab	2.7	0.34

K_D_ of MRU44–4-A1 to α-LTX was tested in different antibody formats (scFv-Fc, IgG, Fab) and on monomeric/dimeric and tetrameric α-LTX with BLI, by supplementing buffer with either 2 mM EDTA or 10 mM CaCl_2_, respectively. IC_50_ values of different MRU44–4-A1 antibody formats were determined in alamarBlue neutralization assay. IC_50_ were not applicable (n.a.) for 2 mM EDTA, as toxin would not be active and thereby have no pathogenic effects on cells and consequently no IC_50_ of antibodies determinable. Resulting values were determined by using Origin Hill1 non-linear fit.

There was no clear trend in enhanced affinity for either monomeric/dimeric or tetrameric toxin. This indicates that the epitope bound by MRU44–4-A1 is accessible in monomeric and multimeric form of α-LTX and is not masked by α-LTX oligomerization. The sensorgrams of the BLI measurements are shown in **Supplementary Figure S1**.

### 3.7 α-LTX neutralization of different antibody formats

**Figure 6:**
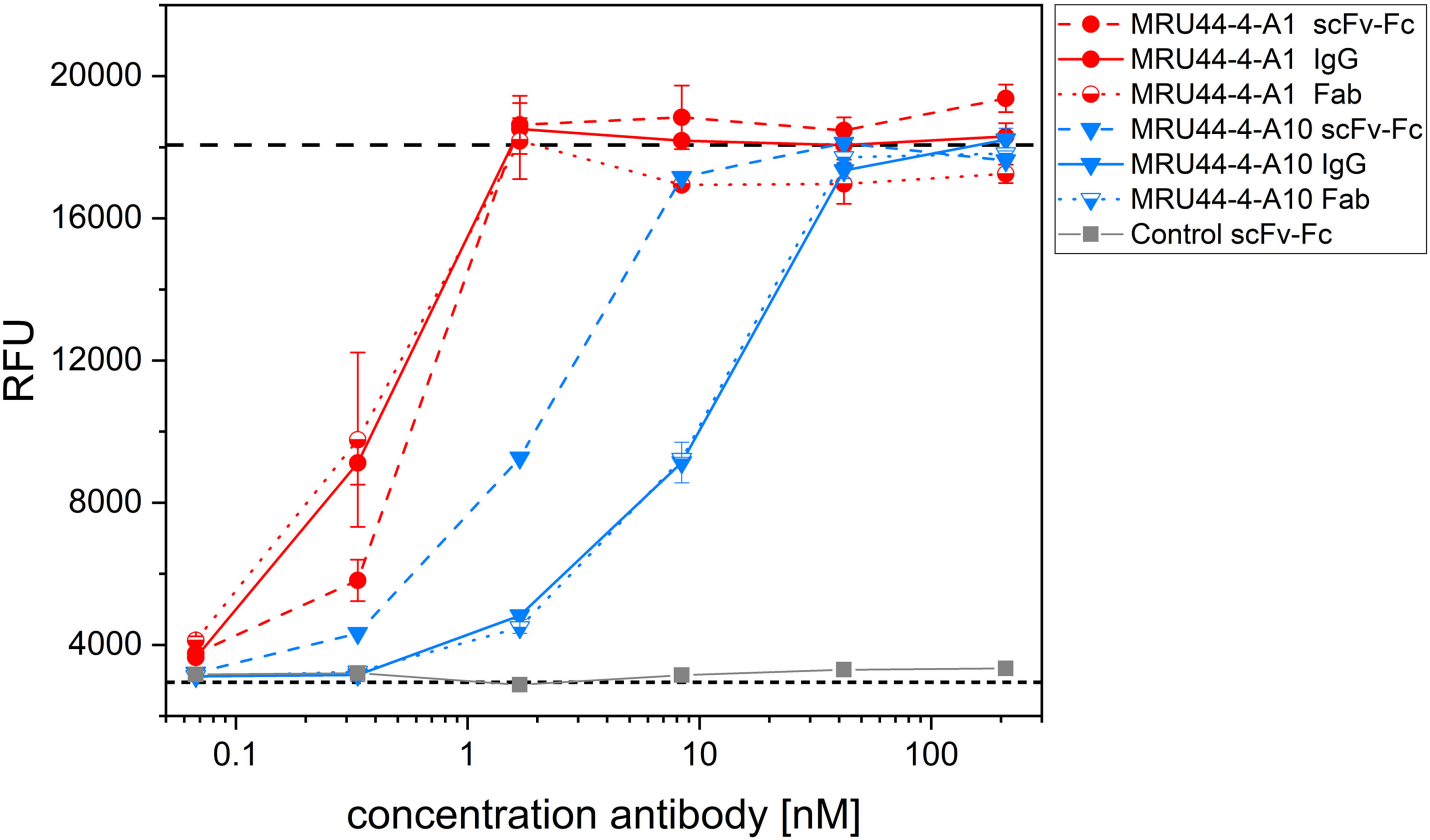
Influence of antibody format on neutralization efficacy. MRU44–4-A1 and MRU44–4-A10 were tested in IgG, scFv-Fc and Fab format in neutralization assay. 210 nM of IgG or scFv-Fc antibody and 420 nM of Fab antibodies were prepared and diluted down to 0.07 nM and 0.13 nM respectively, and incubated with 4.2 nM α-LTX. Used Fab were stoichiometrically corrected to F(ab)_2_ molecules, to compare same quantity of binding moieties. Mean value of mock intoxication, treating the cells without toxin is marked in long dashed lines, mean value of treating cells with α-LTX without adding antibodies is marked in short dashed lines. An unrelated scFv-Fc was included as control. IC_50_ values of different MRU44–4-A1 antibody formats were determined by using Origin Hill1 non-linear fit (see also **Table 2**). Antibodies were tested in duplicates and experiment repeated as a biological replicate with a fresh batch of PC-12 cells. One of the two repetitions is shown as a representative result. Error bars represent the standard deviation of two measurements.

Different antibody formats of the same antibody can influence affinity of antibody-antigen interaction and thereby also influence neutralization efficacy. Furthermore, bivalent antibody formats (scFv-Fc and IgG) in contrast to monovalent formats (Fab) can lead to cross-linking of antigens and thereby cause aggregation/agglutination of the antibody-antigen complexes. The influence of antibody format on the neutralization efficacy was investigated for MRU44–4-A1 and MRU44–4-A10 in the alamarBlue/PC12 cell-based assay (**Figure 6**). Aggregation does not seem to play a role in neutralization, indicated by matching neutralization efficacy and calculated IC_50_ of 2xFab and IgG. In case of MRU44–4-A1, IC_50_ of Fab and IgG was nearly twofold higher than in scFv-Fc. For MRU44–4-A10, scFv-Fc performed considerably better and switch from scFv to IgG/Fab induced a loss of affinity.

### 3.8 Lyophilization to increase shelf-life and quality control

**Figure 7:**
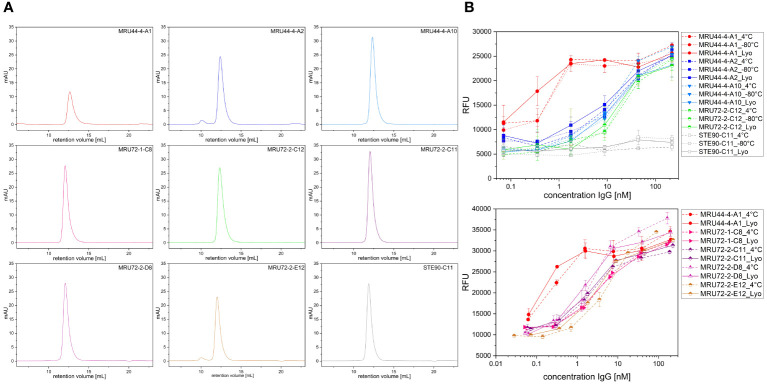
Size exclusion chromatography (SEC) and neutralization efficacy after lyophilization. **(A)** SEC measurement of antibodies selected for lyophilization. Antibodies were reconstituted in ddH_2_O and measured in SEC following lyophilization. **(B)** Neutralization assay of antibodies selected for lyophilization. Antibodies were stored at 4°C and -80°C, respectively. Lyophilized antibodies were reconstituted with ddH_2_O and compared to stored antibody samples. Antibodies were prepared with 210 nM and titrated to 0.07 nM and incubated with 4.2 nM α-LTX. Afterwards cells were intoxicated with toxin:antibody preincubation mix as described. Assay development was done according to screening of lead IgG. Error bars represent the standard deviation of two measurements.

To increase shelf-life of the lead antibody candidates, they were lyophilized according to Schneider et al. (53). 8 antibodies having the highest neutralization efficacy or performing best in titration ELISA or being compatible in combination with MRU44–4-A1 were selected for further development. Samples of antibodies were stored at 4°C and -80°C. Following lyophilization and reconstitution in PBS, size exclusion chromatography (SEC) was used to check for denaturation or aggregation (**Figure 7A**). MRU44–4-A2 and MRU72–2-E12 showed a small fraction of higher molecular weight peak in SEC. All other reconstituted lyophilized antibodies were monomeric. Further, all antibodies tested after lyophilization showed a comparable neutralization efficacy to samples stored at 4°C and -80°C (**Figure 7B**).

### 3.9 Analyzing cross-neutralization with *Latrodectus mactans* venom

Because of the low incidence of black widow spider envenomation, clinical development of a therapeutic antibody is only reasonable if it neutralizes various *Latrodectus* species. Therefore, the aim was to generate antibodies that would cross-neutralize among *Latrodectus* species, which was hypothesized to be likely due to the high sequence identity of Latrotoxins (57). The cross-reactivity of the lead antibodies against α-LTX of the southern black widow (*Latrodectus mactans*) was assessed using whole venom, as purified α-LTX from *L. mactans* was not available.

We tested *L. mactans* venom of two different manufacturers. Here, it became evident, that venom or toxin quality is highly variable and can influence quality of results. Two different lots of each manufacturer were tested and toxicity as well as EC_50_ were matching. The venom of Octolab (VeraCruz, Mexico) was found to be roughly 200x less potent than *L. mactans* venom of Spider Pharm (Yarnell, USA) (**Supplementary Figure S3**). Furthermore, *L. tredecimguttatus* α-LTX was found to be temperature-sensitive and after 3 months of storage at -20°C, nearly no activity could be measured in alamarBlue viability assay (data not shown).

**Figure 8:**
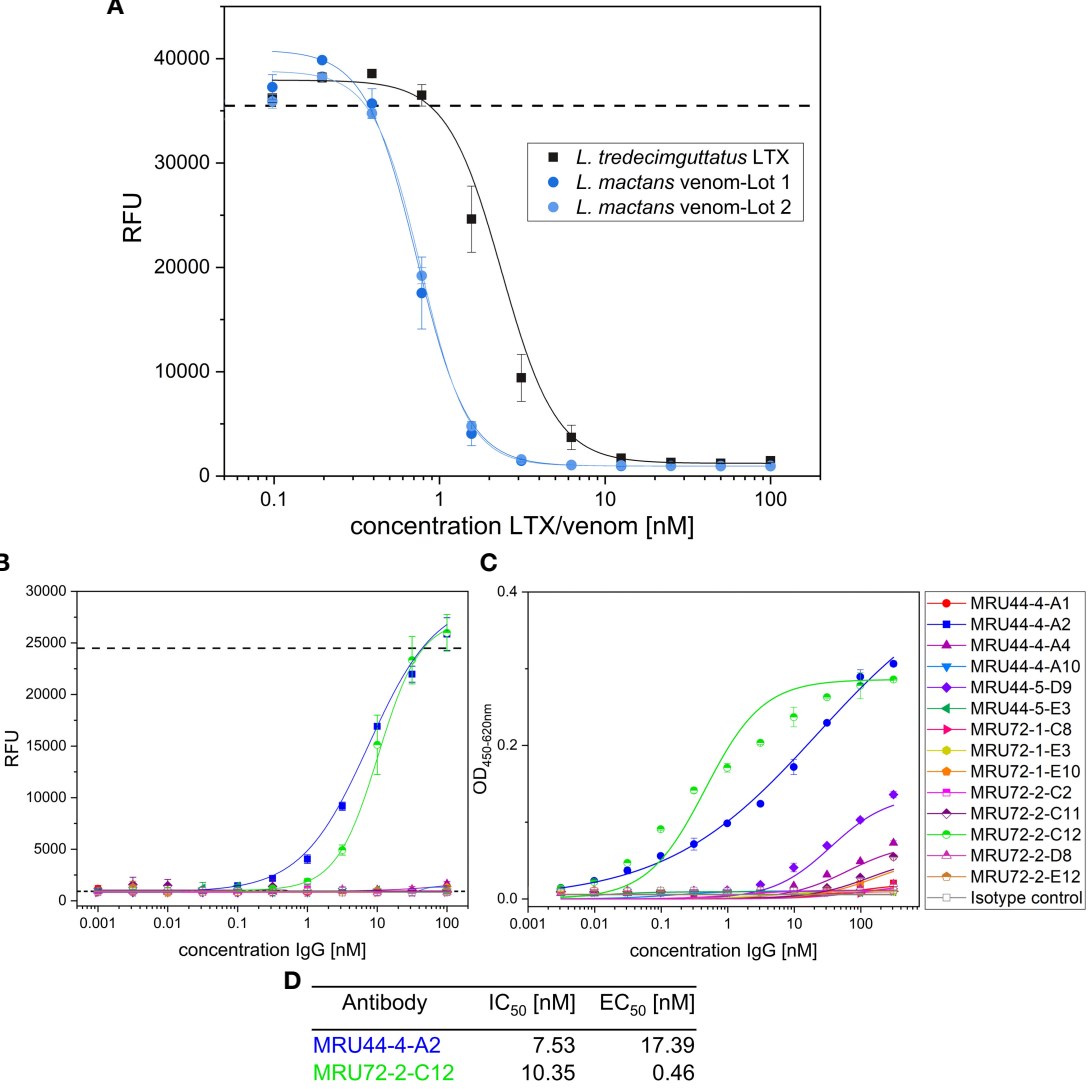
*In vitro* neutralization of *L. mactans* whole venom. **(A)** Comparison of *L. tredecimguttatus* α-LTX to *L. mactans* whole venom. All media were spiked with 10 mM CaCl_2_. Venom/toxin was prepared with 100 nM and diluted to 0.1 nM. 20,000 PC-12 cells/well were intoxicated for 15 h with different toxin/venom concentrations. Mean value of mock intoxication, treating the cells without toxin is shown in dashed line. After intoxication,10% (v/v) alamarBlue was added to the cells and after 6–8 h development emission was measured at 595 nm using Tecan Spark. **(B)** Venom neutralization of *L. mactans* venom with lead IgG. Antibodies were prepared with 100 nM and diluted to 0.001 nM and incubated with 1.5 nM venom for 1 h at RT. Afterwards cells were intoxicated with venom:antibody preincubation mix as described. Mean values of mock intoxication treating the cells without toxin is marked in long dashed lines, mean value of treating cells with α-LTX without adding antibodies is marked in short dashed lines. Assay development was done according to screening of lead IgG. **(C)** Titration ELISA of lead IgG on *L. mactans* venom. 2 µg/mL of venom was immobilized in High binding MTP (Costar) at 4°C overnight. Plates were blocked using 330 µL MPBST and antibody dilutions starting at 310 nM diluted to 0.0031 nM and incubated on venom for 1 h at RT. Staining and measurement of MTP was done as described previously. **(D)** IC_50_ determined in b) and EC_50_ determined in c) by using Origin Hill1 non-linear fit. Error bars represent the standard deviation of two measurements.

For analysis of antibody cross-neutralization between *L. mactans* venom and *L. tredecimguttatus* α-LTX, in a first step the potency of *L. mactans* venom was determined in alamarBlue based cell assay (**Figure 8A**). Despite whole venom being a mixture of different proteins, it was still more potent than purified *L. tredecimguttatus* α-LTX, which was observable in EC_50_ and total signal (lower RFU for saturation of pathogenic effects). After comparing potency of *L. mactans* whole venom to *L. tredecimguttatus* α-LTX, all lead IgGs were tested for their neutralization efficacy against *L. mactans* whole venom (**Figure 8B**). Only 2 out of 14 showed venom neutralization of *L. mactans*. MRU44–4-A1 did not neutralize *L. mactans* venom nor show binding in immunoblotting (**Supplementary Figure S2**). These findings were confirmed by ELISA (**Figure 8C**). MRU44–4-A2 and MRU72–2-C12 showed stronger binding of whole venom compared to all other antibodies tested. The IC50 and EC50 values are given in **Figure 8D**. MRU44–4-A4 and MRU44–5-D9 displayed very faint binding of *L. mactans* venom in ELISA.

## 4 Discussion

The number of recombinant antibodies described against α-LTX is very limited (58–60). Especially mAb 4C4.1 has been thoroughly used in α-LTX research as an affinity reagent (60–62). Due to its murine origin, the potential usage for therapeutic purposes is limited and imposes similar risks for patient safety as animal-derived sera. Furthermore, mAb 4C4.1 was shown to lack cross-neutralization between four different *Latrodectus* species (56). In this study, we therefore developed recombinant human antibodies that neutralize α-LTX and have a potential for the use as a therapeutic alternative to equine antivenom, but also as a diagnostic and research agent. In contrast to standardly used antivenom, human recombinant antibodies are sequence-defined, the production is long-term reproducible in mammalian cell culture and do not exhibit shelf-life issues due to the possibility of lyophilization (31, 53) and represent a non-animal derived alternative (35). Employing different antibody phage display strategies, a total of 75 unique human antibodies binding *L. tredecimguttatus* α-LTX were selected from the naïve libraries HAL9 (lambda)/10 (kappa) (63). Interestingly, despite the high molecular weight of the protein (~130 kDa), four to five consecutive panning rounds were needed to significantly increase the hit-rate. This is in contrast to other antibody selections (41, 46, 48, 52, 64, 65) and most likely explained by a low representation of anti α-LTX scFv in the naïve library. The most efficient strategy was using a mix of both libraries and panning in MTP. Despite the more native display of the antigen in panning in solution or Strep-captured panning approach, only one anti-α-LTX antibody was generated using these approaches. A bottleneck of antibody generation for panning in solution/captured panning on α-LTX could potentially be related to the noticeable size of α-LTX as an antigen or a blocking of essential epitopes by the biotinylation. 53 of the 75 generated antibodies were converted to IgG and produced successfully in mammalian cell culture and 45 of these showed *in vitro* neutralization of *L. tredecimguttatus* α-LTX. Among those, the antibody MRU44–4-A1, showed outstandingly high affinity and neutralization efficacy in all assays tested.

The high rate of neutralizing antibodies indicates that crucial epitopes for α-LTX intoxication were present in naïve conformation and accessible during panning. α-LTX has a hydrophobic base in its tetramerized form (17). Especially the hydrophobic segment 2 (HS2) is well conserved between different Latrotoxins and might be crucial for the mode of different latrotoxins (13, 56). Hereby, antibody selection maybe was driven to epitopes located in the pore-forming (body and wing) area of the protein. The low number of antibodies that bind to other epitopes than MRU44–4-A1 indicates, that there is a structurally dominant epitope.

Using electrophysiology, the high neutralization efficacy of MRU44–4-A1 onto the pathogenic α-LTX-induced increase in neurotransmitter release could be verified in cultured cortical neurons, as it significantly delayed the onset time of α-LTX effects. In three out of 9 single-cell measurements of MRU44–4-A1 and one out of 6 single-cell measurements of antibody combination MRU44–4-A1 and MRU44–4-A2 the 4xSD and thereby onset of relative α-LTX effects was not reached within the measurement period. This means, MRU44–4-A1 efficiently neutralized α-LTX and protected cells from a pathogenic mEPSC frequency increase over measurement fluctuation. MRU44–4-A2 and MRU44–4-A10 showed low neutralization efficacy against α-LTX in electrophysiology, despite their decent neutralization efficacy determined in alamarBlue-based assay. This could be explained due to the lower excess of antibody to α-LTX (10-fold molar excess of antibody to toxin in comparison to 50-fold molar excess of antibody to toxin in highest concentration of alamarBlue assay) and a higher assay sensitivity. The setup was purposely chosen to test for best neutralization efficacy, and to keep it comparable to other monoclonal α-LTX neutralizing antibody studies (59). Synergistic or additives effects of antibody combinations could not be confirmed.

MRU44–4-A1 showed an outstanding neutralization efficacy in comparison to all other antibodies tested with sub-nanomolar IC_50_ of 0.30 nM and EC_50_ tested in ELISA of 0.09 nM, matching results of electrophysiology and BLI. The architecture of antibody and orientation of VH-VL to each other can differ between different antibody formats due to potentially different packing angles of VH to VL (66). Especially when linker-based fusion formats are used (scFv, scFv-Fc), the packing angle can exhibit a broader range in comparison to more rigid packing angles in IgG or Fab (67). Therefore, when switching from scFv to IgG, the packing angle can be altered and affinity potentially be improved or reduced, as described previously (51, 52). Therefore, in our work we switched from scFv to IgG and selected for best-performing antibodies in IgG format, which is also favorable for therapeutics due to well established production, downstream processing and regulatory approval processes. Also, IgG are often more stable and have a longer serum half-life than scFv-Fc antibodies (68, 69). In our case, MRU44–4-A1 showed increased affinity to α-LTX in IgG or Fab format. This was confirmed in alamarBlue neutralization assay and BLI. Furthermore, valency of antibody format can influence antibody binding and thereby neutralization efficacy. Neutralization can be caused by e.g. binding to pathogen or toxin and interfering with the mechanism of invasion, inhibition of protein-oligomerization or inhibition of receptor-binding. In the case of bivalent antibodies, aggregation/agglutination can also be relevant for the mode of action by cross-linking antigens with antibodies (67). In our case, the use of a bivalent antibody format (scFv-Fc or IgG) did not improve neutralization efficacy over monovalent antibody formats (Fab) for MRU44–4-A1 and MRU44–4-A10. This indicates that these antibodies neutralize α-LTX directly, e.g. by blocking oligomerization, impairing membrane insertion, or directly blocking α-LTX pore in the tetramerized form.

As latrodectism is especially a health issue in tropical countries, that may lack a constant cooling chain, lyophilization offers improvement of long-term storage, which can be problematic for products stored in liquid formulation at ambient temperatures (70). None of the antibodies showed degradation after lyophilization and only MRU44–4-A2 and MRU72–2-E12 showed a small fraction of higher molecular weight complexes. Nevertheless, none of the antibodies tested showed impaired neutralization efficacy after lyophilization, including MRU44–4-A2 and MRU72–2-E12, which suggests eight potential candidates for future development.

An important observation was the highly varying venom quality of the same *Latrodectus* species (*L. mactans*) obtained from different manufacturers. Venom from Octolab was more than 200-fold less potent than venom obtained from Spider Pharm and, at least 70-fold less potent than purified *L. tredecimguttatus* α-LTX obtained from Alomone Labs. The comparison of *L. tredecimguttatus* α-LTX and *L. mactans* venom obviously is only partly possible, as different *Latrodectus* species are known to have different venom potencies. Nevertheless, the differences are usually in a significantly smaller range (57, 71). The toxin/venom is very sensitive to external stressors (e.g. temperature observed in stability tests with *L. tredecimguttatus* α-LTX). Therefore, we recommend to always implement internal quality control assays, such as the alamarBlue assay described herein, to make sure to have decent venom material, before going for venom or toxin studies.

Out of 14 antibodies, only MRU44–4-A2 and MRU72–2-C12 showed cross-neutralization to *L. mactans* venom, while being selected on *L. tredecimguttatus* α-LTX, indicating conserved epitopes between the α-LTX species for these antibodies. All other antibodies did not cross-neutralize while a few retained at least weak binding in ELISA. This was expected, as the epitope binning showed that all antibodies except for those two bind to an overlapping epitope area to MRU44–4-A1. Evidently this epitope area must be in a non-conserved region between *L. tredecimguttatus and L. mactans* α-LTX. On the other hand it was surprising that so many antibodies were selected in the same epitope area. Furthermore all *Latrodectus* species show high protein identity for α-LTX and the literature described α-LTX as very similar between the different black widow species (12). To be noted, full-length sequences of α-LTX from different *Latrodectus* species only exists for a few widow spiders (e.g. *L. tredecimguttatus, L. hesperus, L. hasselti*) and proposed sequence identity is based on 618 bp long PCR fragments (12). If sequence identity would be correct for all full-length α-LTX from widow spiders, it is still possible that there are more structural differences than known to date, as only *L. tredecimguttatus* α-LTX structure was thoroughly analyzed by Cryo-EM so far (17). Føns et al. described the first recombinant human IgG generated by phage display, which was also selected on *L. tredecimguttatus* α-LTX (59). The authors hypothesized that the antibody should be cross-neutralizing between *Latrodectus* species, due to the high-sequence identity of α-LTX. In line with our findings and the research done on mAb 4C4.1 (56), we question this hypothesis.

For therapeutic purposes a lack of cross-neutralizing efficacy is a disadvantage and equine-derived antivenom often is described to show cross-neutralization between *Latrodectus* species (45, 57, 57, 72, 73). Nevertheless, due to the high sequence specificity, MRU44–4-A1 and other non-cross neutralizing antibodies could be used for diagnostic purposes and as research tools, to discriminate between α-LTX of different *Latrodectus* species. Furthermore, for therapeutic purposes, an oligoclonal antibody combination (45) of different antibodies (e.g. MRU44–4-A1 combined with MRU44–4-A2) mimicking a polyclonal antibody mixture could be used, to target different α-LTX, if species origin following envenomation is unknown. To facilitate production and regulatory approval, MRU44–4-A1 could be engineered as a bispecific antibody, combining different binding moieties specific for different α-LTX, as agglutination does not seem to be crucial for α-LTX neutralization.

Due to the limited availability of α-LTX full-length sequences and structural data, we propose the generation of multiple antibodies against α-LTX to ensure broad cross-reactivity against different *Latrodectus* species. Antibodies described here are outstanding for their exceptionally high affinity to *L. tredecimguttatus* α-LTX in sub-nanomolar and nanomolar ranges. Furthermore, we showed cross-neutralization for at least two antibodies generated between *L. mactans* and *L. tredecimguttatus.* Lastly, antibodies generated here can be improved to increase affinity against other *Latrodectus* species, e.g. by affinity maturation (64, 74).

These antibodies could be a future replacement for animal-derived antivenom, and they could be used in diagnostic and as research tools.

## Data availability statement

The original contributions presented in the study are included in the article/**Supplementary Material**. Further inquiries can be directed to the corresponding author.

## Ethics statement

Ethical approval was not required for the studies on humans in accordance with the local legislation and institutional requirements because only commercially available established cell lines were used. All animal procedures were approved in advance by the federal Saxonian Animal Welfare Committee (T01/21 for mice). The study was conducted in accordance with the local legislation and institutional requirements.

## Author contributions

MR: Conceptualization, Data curation, Formal analysis, Writing – original draft, Writing – review & editing, Investigation, Methodology, Visualization. JN: Data curation, Formal analysis, Investigation, Methodology, Writing – review & editing. MB: Investigation, Writing – review & editing. DM: Investigation, Writing – review & editing. SP: Investigation, Writing – review & editing. KC-L: Conceptualization, Investigation, Writing – review & editing. PK: Formal analysis, Investigation, Writing – review & editing. AL-N: Conceptualization, Funding acquisition, Methodology, Project administration, Supervision, Writing – review & editing. SH: Formal analysis, Supervision, Writing – review & editing. SD: Formal analysis, Supervision, Writing – review & editing. MS: Formal analysis, Investigation, Methodology, Supervision, Writing – review & editing. JB: Funding acquisition, Project administration, Supervision, Writing – review & editing. MH: Conceptualization, Data curation, Formal analysis, Funding acquisition, Methodology, Project administration, Supervision, Writing – original draft, Writing – review & editing.
