# Supplementary material for: Human antibodies neutralizing the alpha-latrotoxin of the European black widow
Source: Front Immunol. 2024 Jun 12;15:1407398. doi: 10.3389/fimmu.2024.1407398 (PMC11199383; doi:10.3389/fimmu.2024.1407398)
Supplement: Supplementary file 1 [file DataSheet_1.pdf]

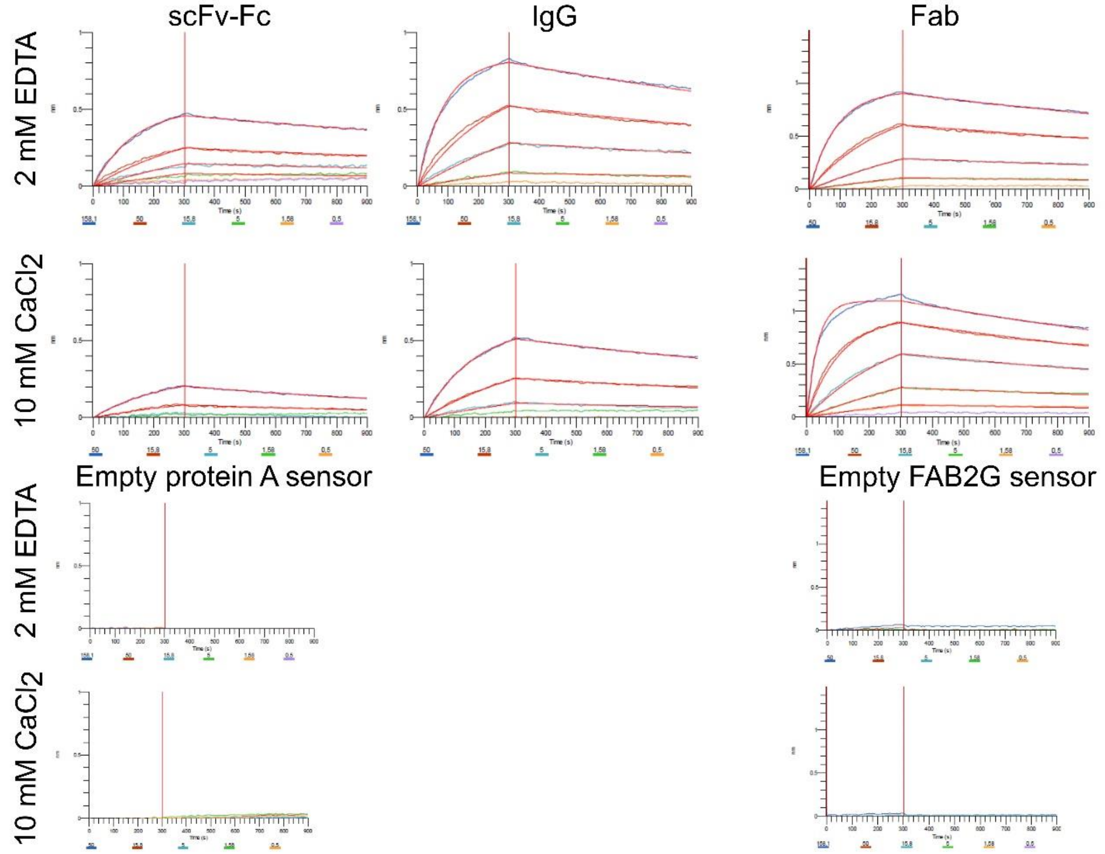

Supplementary Figure S1: BLI sensorgrams of MRU44-4-A1 in different antibody formats. Antibody affinity of MRU44-4-A1 to α-LTX was measured in scFv-Fc and IgG format using ProA biosensors and in Fab format using FAB2G sensors. Measurements were done in presence of 10 mM $CaCl_2$ to induce oligomerization of α-LTX, or in presence of 2 mM EDTA to block oligomerization of α-LTX. Baseline measurement was performed for 60 s before antibodies were loaded onto the sensors for 180 s with 10 µg/mL of antibody. After establishing a stable baseline, association of α-LTX was measured for 300 s in dilution series from 158 nM to 0.5 nM. Dissociation of α-LTX was measured for 600 s. Controls without α-LTX and with unloaded/empty sensors were included. Analysis was done by substracting the baseline measurement (0 nM α-LTX) and modelling the binding kinetics was done with a global 1:1 binding model.

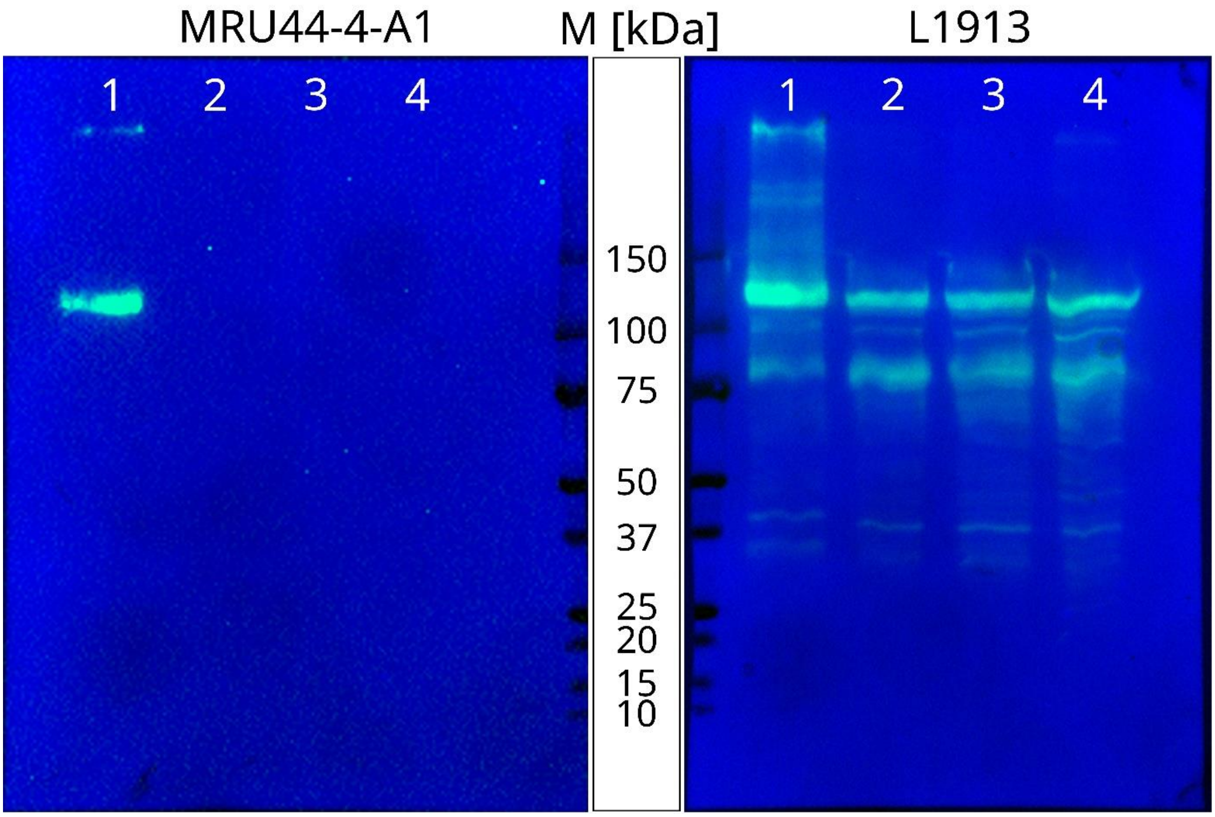

Supplementary Figure S2: Immunoblotting of *L. mactans* venom and *L. tredecimguttatus* α-LTX. Always 2 µg *L. tredecimguttatus* α-LTX from Alomone Labs (1), *L. mactans* venom from Spider Pharm (2,3) and *L. mactans* venom from Octolab (4) were separated in 8%-12% SDS PAGE and transferred to PVDF membrane for immunoblotting. M = Precision Plus Protein™ All Blue Standards. Detection was done using 10 µg/mL MRU44-4-A1 primary antibody and A0170 (anti-human-IgG-HRP, final dilution 1:70,000) secondary antibody (left blot), or L1913 (rabbit-anti-α-LTX, final dilution 1:50) primary antibody and donkey-anti-rabbit-IgG-HRP (Jackson-Immuno-Research, 711-035-152, final dilution 1:20,000) (right blot) using SuperSignal™WEST Pico PLUS Kit. Channel for chemiluminescence and bright field were merged using ImageLab.

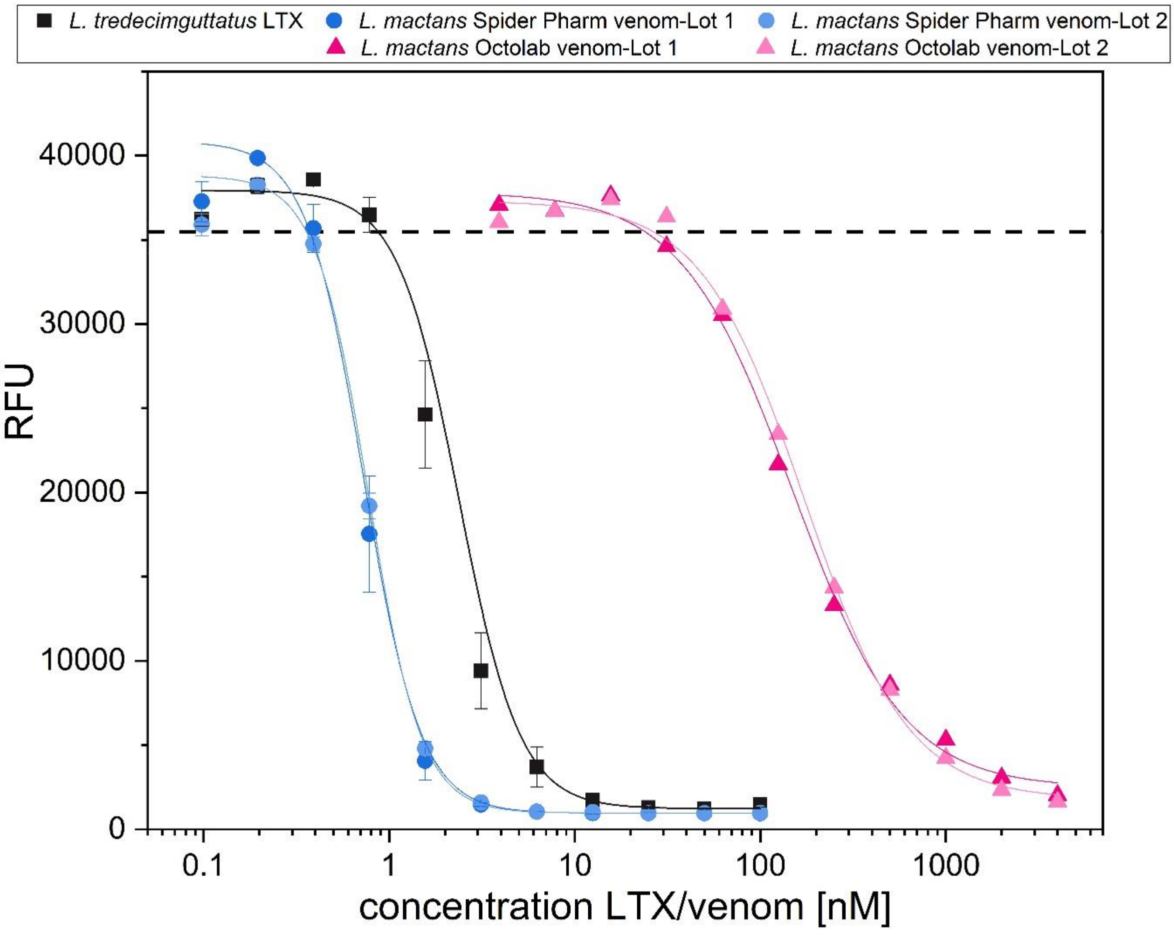

Supplementary Figure S3 Comparison of *in vitro* activity of two different manufacturers for *L. mactans* whole venom preparations with *L. tredecimguttatus* α-LTX. All media were spiked with 10 mM $CaCl_2$. Venom/toxin was prepared with 100 nM and diluted to 0.1 nM in case of Alomone Labs and Spider Pharm toxin/venom, while Octolab venom was prepared with 4000 nM and diluted to 3.9 nM. 20,000 PC-12 cells/well were intoxicated for 15 h with different toxin/venom concentrations. Mean value of mock intoxication, treating the cells without toxin is shown in dashed line. After intoxication,10 % (v/v) alamarBlue was added to the cells and after 6-8 h development emission was measured at 595 nm using Tecan Spark. Error bars represent the standard deviation of two measurements.
